# Abstract of the Proceedings of the Buffalo Medical Association

**Published:** 1858-05

**Authors:** Austin Flint


ART. II.—*Abstract of the Proceedings of the Buffalo Medical Association.*

TUESDAY EVENING, March. 2, 1858.

The Association met.

Present—The President, Prof. Flint, in the chair; Drs. Wyckoff, Hamilton, White, Strong, Wilcox, Mixer, Hawley, Eastman, Rankin, Rochester, Gay, Root, Newman, Ring, and Flint, Jr.

The minutes of the last meeting were not present, and on motion, their reading was dispensed with.

*Dr. Newman* then read his report on Puerperal Convulsions.

*Report of the Committee on the connection of Albuminuria with the development of Puerperal Convulsions; and the employment of Chloroform as a remedial measure.* By JAMES M. NEWMAN, M. D., Chairman of the Committee.

GENTLEMEN:

To your committee was assigned the duty of collating and presenting such facts as have a bearing upon the etiology of Puerperal Convulsions; especially in reference to the influence of the condition of *albuminuria* in predisposing and developing these convulsive seizures in the pregnant female; together with the comparative value of the employment of anæsthetic agents in addition to, or as a substitute for the usual treatment of copious venesection, or other depleting measures.

Puerperal eclampsia is, fortunately, a comparatively rare complication of gestation and parturition, and the opportunities for the study of the disease in the usual obstetrical practice of medical men infrequent, and the etiology of the affection is, we may conclude, to be only slowly evolved.

Dr. Arneth, among 6527 labors observed 13 cases of eclampsia, or 1 in 502.*

Dr. Carl Braun records the result of his own experience in the observation of 52 cases of convulsions occurring in 24,132 labors: 1 in 464.†

Dr. F. H. Ramsbotham, in the obstetrical statistics of the Eastern District of the Royal Maternity Charity, embracing a period of twenty-four years, and comprising 55,182 labors, reports 55 cases of puerperal convulsions, or 1 in 1003.3.‡

Dr. Churchill gives the statistics as collected from several authorities, where 273 cases of convulsions occurred in 190,313 labors, or 1 in about 693.75.§

Out of 20,357 labors under the care of the late Madame Boivin, at the Maternité, at Paris, the number of patients affected with eclampsia, was 19, or 1 in every 1071½.

Mad. Lachapelle, in 38,000 labors, had 61 cases of convulsions, or 1 in 622.

In the report of the Edinburgh Royal Maternity Hospital, for the period of two years, from 1844 to 1846, are recorded 1475 labors in the care of that charity, in which there were 4 cases of convulsions, or 1 in 354.‖

Cazeaux, in two thousand deliveries occurring under his care, at Hôtel Dieu and the hospital of La Faculté, met with but three cases of convulsions: 1 in 666⅔.¶

The testimony of American obstetrical statistics upon this subject, as far as we have been able to collect them, is as follows:

Dr. James C. Bliss, in 820 cases of labor, encountered four cases of convulsions, or one in every 205 births.**

Dr. John Geo. Metcalf, of Massachusetts, in a statistical report of 927 cases of labor, reports 2 cases of convulsions, or 1 in 463.5 labors.††

Dr. H. Pleasants, of West Philadelphia, in 420 labors, had two cases of convulsions, or one in every 210 labors.‡‡

---

* British and Foreign Med. Chirurg. Rev., Oct., 1851, p. 442.

† British and Foreign Med. Chirurg. Rev., Jan., 1855, p. 40.

‡ Process of Parturition. By F. H. Ramsbotham, M. D. Philadelphia: 1855. pp 505–512.

§ Churchill's Diseases of Women. Philadelphia: 1857. p. 710.

‖ Simpson's Obstetric Memoirs, vol. i., p. 754.

¶ P. Cazeaux's Midwifery. Am. Ed. Philadelphia: 1857. p. 715.

** New York Journal of Medicine, Jan., 1847.

†† American Journ. Med. Sciences, Oct., 1847, p. 304.

‡‡ Am. Journ. Med. Sciences, April, 1848, p. 366.

Dr. D. Humphreys Storer, in a report from the Boston Lying-in-Hospital, describes the particular points of interest in 451 cases of labor, and among which convulsions occurred in three instances, or once in 150.33 labors.*

Dr. John P. Little, of Richmond, Virginia, in 45 cases of labor, met with three cases of convulsions, a proportion much higher than any of the statistics we have yet given.†

Dr. Daniel Pierson, of Augusta, Ill., in the statistics of 274 obstetrical cases which he has preserved, and given to the profession, details four cases of convulsions, or one in every 68½ cases of delivery.‡

Dr. Walter Channing, of Boston, in his work on Etherization in Child Birth, has collected from his own experience, as well as from the communications made to him by his professional brethren in Boston and vicinity, 574 *chloroform cases*, in which 10 cases of *convulsions* occurred, or 1 in 57.4. As these are select cases, given for the purpose of establishing the safety and utility of anæsthetics in labor, they, perhaps, ought not to be taken as any basis upon which to found a numerical calculation of the frequency of convulsive seizures; but at the same time, the limited period in which these observations were made, can scarcely fail to suggest to the mind a computation of the comparative frequency of this complication of the puerperal state.

The most common subjects of puerperal convulsions are women in their first pregnancies, the strong and healthy, perhaps, being the most liable.

---

* Am. Journ. Med. Sciences, Oct., 1850, p. 361.

† Am. Journ. Med. Sciences, Jan., 1851, p. 87.

‡ Am. Journ. Med. Sciences, July, 1857, p. 59.

NOTE. So far as the few American statistics which we have been enabled to collect upon this subject, prove anything, we fear that the evidence which they bear is, that puerperal convulsions are much more frequent in this country than in Europe. It is a subject not unworthy of the future study and notice of obstetrical writers, whether such be the fact, and to endeavor to discover, if possible, whether anything in our national habits, or physical, or mental peculiarities contribute to the predisposition of this complication of the puerperal state. We think that an examination of the reported cases would very plainly intimate that the disease for the past few years has been much more frequent than usual, if it be not upon the increase. In making a comparison, however, upon this point, the fact should not be allowed to pass unnoticed, that the European statistics are generally gathered from hospital practice, with its corresponding large aggregate of numbers; while our American statistics are from private practice almost exclusively, and perhaps, in a majority of cases, have occurred in the *consultation* practice of the reporters, which charges to their account, the cases occurring in their own practice and in that of their friends, and which would very materially add to the per centage of frequency.

Many of the subjects, too, are among the unmarried, a fact not unworthy of notice, as indicating a probable connection between the mental emotions of shame, grief and all those other sources of nervous disquietude from which the pregnant unmarried woman suffers, and the subsequent development of that intense disturbance of the nervous centres necessary to the production of a fit of eclampsia. The liability of primiparæ to puerperal convulsions, is abundantly proven by statistics, and is admitted by all observers of the disease.

Dr. Arneth, among 13 cases, indicates 8 as first pregnancies.*

Of 12 cases reported by Mr. Rose, 9 were primiparæ and 8 unmarried. Of the remaining 3, 1 was a third pregnancy, and the other 2 were twelfth pregnancies.†

The statements of Blot and Litzmann, exhibit the presence of albuminuria in 56 primiparæ out of 178, and in 22 multiparæ out of 159.‡

Prof. Simpson, in some published statistics, bearing upon this point, gives 233 cases of puerperal convulsions, occurring in the practice of different medical men, in which 178, or 76 per cent. happened in first labors.§

The seasons of the year appear to exert little, or no influence in the production of these convulsions, and females are attacked indifferently in all the months of the year.

As a general rule, puerperal convulsions are quite rare previous to the sixth month of pregnancy. As examples of the occasional early complications of convulsions, Cazeaux‖ relates the following cases:

"M. Danyau, Sr., however, met with it in a young girl, who had only reached the sixth week, and in whom nothing but the extraction of the ovum could remove the symptoms. The eclampsia came on again in her next pregnancy, about the same period, and was followed by abortion; but in this instance, the fits continued for some time after abortion.

"A lady of Ferrara, about twenty-eight years of age, of a bilious temperament, and the mother of three children, was periodically attacked by convulsions as soon as she had conceived, and these attacks were renewed every two weeks through gestation; so that their appearance constituted in her a sign of pregnancy."

---

* British and Foreign Med. and Chirurg. Review, Oct., 1851, p. 442.

† Med. Times and Gazette, July 17, 1852, p. 61.

‡ Brit. and For. Med. Chirurg. Review, Oct., 1855, p. 427.

§ Simpson's Obstetric Memoirs, vol. i, p. 386.

‖ P. Cazeaux's Midwifery. Second American Edition. 1857. p. 715.

Authors, as a general rule, have divided puerperal convulsions into three forms, the *hysteric*, the *epileptic* and the *apoplectic;* and have described the symptoms of each form with all that minute exactness, as if it were possible to make these hair-breadth distinctions at the bed-side, or as if their subsequent inculcations for practice were to be particularly governed by the ability to diagnose a particular form of convulsions.

We confess to our skepticism as to the ability of the physician to make these nicely drawn distinctions in the convulsions of the pregnant woman—and the uniformity of practice in the treatment of these cases which has heretofore attained among the profession, would seem to indicate, either the impossibility of making such distinctions, or else, that one form of treatment is applicable to all cases, and that no necessity, as a consequence, existed for the establishment of a differential diagnosis.

We cannot, therefore, but regard the divisions of the forms of this disease as adopted and taught by Dr. Murphy,* as more rational, and more in accordance with the true pathological conditions of the system at the time of the development of the convulsions, and as furnishing a better index to the indications for treatment, than the arbitrary adoption of an arbitrary name, which calls as arbitrarily for a peculiar and established form of treatment: or, in other words, we believe the adoption of the latter division of the disease, will be more likely to lead the physician to treat *symptoms* and *conditions* rather than *names.*

Dr. Murphy has divided this class of affections under the subdivisions of *sthenic*, or *hyperæmic; asthenic*, or *anæmic;* and *hysterical* convulsions.

This arrangement indicates the opposite conditions of the system in which convulsive phenomena may be exhibited, and suggests at once the very different forms of treatment which may be needed to meet each individual case

It is time the term *apoplectic* was dismissed from our nomenclature, for our advanced state of pathology, has long since taught, that to be *apoplectic* is not necessarily to be *hyperæmic;* and that death may ensue as readily upon an *anæmic* condition of the system, and a want of a due and proper stimilus of the brain, as upon a condition of congestion of that organ.

It is not uninteresting, in studying the literature of puerperal convulsions to notice, how the writers have endeavored to preserve the peculiar diagnostic phraseology of the several forms of the disease as laid down in the books, and how far their treatment has after all, been governed by the actual con-

---

* Lectures on the Principles and Practices of Midwifery. By Edward W. Murphy, A. M., M. D. London: 1852.

dition of their patients. We think a commentary, upon the valueless character of an arbitrary nomenclature, even if it be sanctioned by venerable names and by those high in authority, might be easily framed from the evidence of these recorded cases, where the rational signs of each case have been met by a rational form of treatment, irrespective of the peculiar bias impressed upon the physician by preconceived pathological views and opinions.

The divisions which have been made in the classification of the several forms of this disease, to a certain extent, indicate the pathological views extant in reference to the causes of its production.

To a condition of congestion, or hyperæmia of the cerebral organs has been almost universally assigned the production of the convulsions peculiar to, or attendant upon, the puerperal state.

We do not deem it necessary to cite authorities upon this point. We think there will be no dispute of the truth of the assertion.

It is, perhaps, not unworthy of notice, that this view has been taken and accepted from the *symptoms* exhibited *during the life* of the patient, and while suffering under the convulsive seizures, rather than from *post-mortem* revelations of injuries inflicted upon the brain and nervous centres, or from the discovery of an actual state of congestion. Authors agree, that examinations *after death* throw little or no light upon the true pathology of this disease, that there are generally no traces left, or evidences furnished, as to the organs involved, and reäcting, in the production of the convulsions. *The nervous centres at least, are no more freguently the seat of changes, and of organic lesions*, than are the other organs of the body.

We refer more particularly to the theory of congestion, from the important part it has had in directing and upholding the treatment practiced for the relief of these convulsive seizures.*

We now pass in the consideration of this subject, to a period when more correct views of the causes involved in the development of puerperal convulsions, and of the peculiar pathological conditions of the system under which they are developed, would seem to have been evolved.

Puerperal convulsions are now thought to be generally, though not

---

* We would refer those who are curious to trace out the pathological views, until lately held in reference to this subject, and who wish to enter into an examination of their soundness, to the work entitled "Parturition and the Principles of Obstetrics. By W. Tyler Smith, M. D., London. Published by Lea & Blanchard, Philadelphia. 1849." Lectures xx and xxi.

always, the result of changes in the blood, and produced in consequence of the toxicological effects of such alterations in the normal character of the circulating fluid; and that such blood-poison follows upon the non-elimination of some of its usual excretions.

Attendant upon this condition of faulty secretion, or excretion, as its early and constant sign, is the presence of *albumen* in the urine, and while such excretion takes place from the kidneys, there is retained within the circulation compounds usually eliminated by these organs, and of which the principal one is, probably, urea.

To this condition of the excretion of albumen, the term has been given of *albuminuria;* and to the symptoms especially chargeable to the blood-poison, and derived from the hypothetical part assigned to the retained urea, *uræmia*, or *uræmic intoxication* has been applied; and the latter term may, perhaps, be now regarded as a generic name applicable to, and covering all the symptoms growing out of this peculiar condition of the system.

We do not propose to discuss any of the questions, which a recognition of this condition has suggested in the pathological search after causes and the peculiar modes by which these impressions are expended upon the organism. Whether the poisoning is in fact occasioned by the retained urea, is a disputed question.

That a certain set of symptoms follow a condition of *albuminuria* is a well established fact, and from being generally referable to disease of the kidneys, has constituted a subject of study which has been most ardently pursued. And among the results of the investigations made to elucidate these points, not the least valuable has been the knowledge which has been evolved in reference to the causes concerned in the development of convulsions in the pregnant woman.

For more than fifty years the important fact has been noticed by authors, that there was a tendency in pregnant women, the subjects of anasarcous swellings, especially of the upper extremities and face, to suffer from convulsions. This fact has been alluded to, and confirmed by the observations of Hamilton, Demanet, Duges, Burns, Montgomery, Ingleby, Johns and others. But they seem to have deduced no important conclusions from these observations, nor to have so connected them in their probable relations of cause and effect, as to render them valuable as means of diagnosis and the basis of a course of treatment, which should not only be instituted in the hour of actual suffering from this most terrific of all the accidents of the gestative and parturient state, but which should lead us to so far anticipate the probabilities of a convulsive seizure, that a course of preventive treatment might be instituted which should aim to guard the patient from the impending fate which awaits her.

Prof. Simpson, of Edinburgh, seems to have been the first to recognize distinctly the conditions under which the state of anasarca led to, or predisposed to an attack of convulsions during the period of gestation, or in the act of labor; and to direct the profession in a course of investigations, the results of which, when we consider the important ends which they promise to serve in directing our treatment, not only as curative, but preventive of the convulsive seizures, deserve to be placed in foremost rank among the most valuable of the numerous additions lately made to our stock of pathological knowledge.

We deem the subject worthy of distinct record in this connection, of the successive steps by which a knowledge of these facts has been arrived at, and of according that credit to the several observers to which they are justly entitled, preserving the chronological order of their labors, so for as the limited number of authorities at our command will enable us to do.

Prof. Simpson says,* "Previously to my first course of lectures on Midwifery, in the University of Edinburgh, in 1840–1, I had, in more than one case, ascertained the œdema or anasarca seen in patients affected with puerperal convulsions, to be one of the numerous and important forms of dropsical disease which Dr. Bright had shown to be connected with the existence of albumen in the urine. Up to 1843 I had detected, in repeated instances, this connection of puerperal convulsions with albuminuria, but hitherto I had found it only by examination of the urine during life. In the spring of 1843 I saw a fatal case of puerperal convulsions, in which, in addition to the detection of albuminuria during life, I had an oppertunity of observing the usual granular disease of the kidney on post-mortem examination.

"This case offered me the first opportunity of confirming, by inspection after death, an opinion that I had been led to adopt from the examination of the symptoms during life, and had publicly taught for the last two sessions, viz: that patients attacked with puerperal convulsions had almost invariably albuminous urine, and some accompanying, or rather preceding dropsical complication, and hence probably granular renal disease.

"My friend Dr. Lever, of London, who happened also to be directing his attention at the same time to the connection of puerperal convulsions with albuminuria, published in the number of Guy's Hospital Reports for 1843,

---

* Simpson's Obstetric Memoirs and Contributions, pp. 726–7.

several cases of puerperal eclampsia, in five of which he had found the urine to be albuminous.

"Since the above date (1843) I have seen a variety of cases of puerperal convulsions in consultation and hospital practice, and have always (with very rare exceptions indeed) detected the existence of albuminuria in the urine of the mother. In one or two instances I have found the kidneys presenting traces of recent acute inflammation; as pus, &c. Sometimes convulsions, or symptoms threatening them, recur in successive labors in the same mothers in connection with established granular disease. Usually, however, the state of albuminuria which leads to puerperal convulsions, is a transitory morbid condition, from which the patient recovers within the course of a few days after delivery; and the affection does not depend upon, or result in, any actual change of structure of the kidneys."

Dr. Lever prosecuted his investigations with such zeal, that in 1844,* he had remarked that in nine cases out of ten of puerperal convulsions that albuminous urine existed. He tested the urine of more than fifty women, drawn during labor with great care with the catheter, and the result was, "that in no case was albumen detected except where there had been convulsions, or their recognized premonitory symptoms. Urea was sought for in the blood in one instance, but not detected."

In 1847, Prof. Simpson,† in a communication upon the same subject, the result of further observation, draws the following conclusions:

"1. Albuminuria, when present during the last periods of pregnancy and labor, denotes a great and morbid *tendency* to puerperal convulsions.

"2. Albuminuria, in the pregnant and puerperal state, sometimes gives rise to other and more anomalous derangements of the nervous system without proceeding to convulsions; and I have especially observed states of local paralysis and neuralgia in the extremities, functional lesions of sight (amaurosis, &c.,) and hearing; hemiplegia and paraplegia more or less fully developed.

"3, Œdema of the face and hands, going on occasionally to general anasarca, is one of the most frequent results of albuminuria in the pregnant female.

"4. The presence of œdema (3), or of any of the lesions of the nervous system (2), with or without the œdema, should always make us suspect albuminuria; and if our suspicions are verified by the state of the urine, we

---

* Braithwaite's Retrospect. Part x, p. 76.

† Simpson's Obstetric Memoirs and Contributions, vol. i, p. 733.

should diligently guard, by antiphlogistic means, &c., against the supervention of puerperal convulsions."

Dr. Mickschick* tested the urine of 26 women in labor, and detected albumen in the urine of five patients, one of whom was dropsical. Two women were attacked with convulsions, but albumen was found in the urine of but one of them.

M. Labatt has reported an interesting case where puerperal convulsions came on in a highly anasarcous woman, who a tlength sank into a comatose condition. By degrees, as the anasarca diminished under the influence of remedies, the albumen, with which the urine had been loaded, disappeared, and the intellectual powers returned.

In 1847, MM. Devilliers and Regnault,† presented to the Academy of Medicine of Paris, the result of their investigations upon this subject. They state that

"In this condition the affection presents much less distinct and well defined symptoms, most of which may be readily confounded with those of other diseases of pregnany. Sometimes no morbid phenomenon manifests itself; and at others there may be heaviness, headache, general uneasiness, some characteristicless derangements of the digestive organs; but no febrile action belonging to this disease in particular. Lumbar pains are so common in pregnancy, that we are unable to distinguish them either by their seat or nature from those which are developed during the acute stage of albuminuria. * * * * * * "The affection which especially presents itself as a frequent and important complication of albuminuria is eclampsia. Most of the subjects of puerperal convulsions exhibit evident marks of serous infiltration. In some, however, this symptom is absent; but the authors of this memoir have *constantly* found albumen in their urine. Of course *all the women exhibiting albumen do not become the subjects of convulsions;* and the authors have found these absent eight times in twenty cases; but they believe that more than a mere coincidence exists between the two diseases, founding their opinion upon the fact, that in ordinary albuminuria various cerebral and nervous phenomena are exhibited, and that during pregnancy these last acquire a special development.

"Contrary to what is observed in ordinary cases, the albuminuria of pregnancy may terminate in a rapid and complete cure after confinement, how-

* British and Foreign Medical Review, Jan., 1847, p. 289.

† Med. Chirurg. Rev., July, 1847. Quoted from Amer. Journ. Med. Sciences, Oct. 1847, p. 505.

ever grave the case may have appeared. It may pass into the chronic state, but this is more rare. * * * * * *

"The authors observed 11 deaths in 20 albuminuric women. * * *

"They feel much disposed to accord to organic lesions much less importance than is ordinarily attributed to them, and and to regard them rather as the effects than the causes of the disease. Indeed, in pregnant women its causes seem to be different from those generally admitted in ordinary albuminuria.

"The researches of these authors have proved to them that the blood of women during gestation exhibits a remarkable diminution of albumen, especially in the latter months; a condition which Andral has shown to be favorable to the production of dropsies in general, and one which bears the relation of cause to effect in respect to the present disease."

May 12th, 1849, at a meeting of the *Westminster Medical Society*, Dr. Cormack* read a paper detailing three cases of puerperal convulsions which had occurred in his practice. The main object of the paper was to point out the connection which exists, in a very great proportion of cases, between renal congestion and puerperal convulsions.

He considered puerperal convulsions to be — though not always, yet generally — the toxicological results of non-elimination of the excretions of the blood; and that, in by far the greater number of cases, this non-elimination depends on renal congestion, caused by the pressure of the gravid uterus. Œdema and albuminuria are frequent concomitants or precursors of convulsions, as shown by Dr. Lever and MM. Devilliers and Regnault.

Among the twelve cases of puerperal convulsions related by Mr. Rose, five had been preceded by albuminous urine.

Dr. Hecker† relates three cases of albuminuria in pregnant women, two of which had convulsions during labor. One proving fatal soon after delivery, and the other recovering rapidly. The *albuminous* patient, having *no convulsions*, presented thirty-six hours after delivery her urine clear, neutral and without a trace of albumen. She died three weeks after delivery, of peritonitis. The kidneys were pale, flabby and bloodless, and easily separated from the capsules; their tissue exhibited no change under the microscope.

Guy's Hospital Report, 1856,‡ contains the notices of twenty-seven cases

---

* Amer. Journ. Med. Sciences, April, 1850, p. 530. From London Journal Medicine, Nov., 1849.

† Brit. and For. Med. Chir. Review, July, 1854. p. 205.

‡ Guy's Hospital Reports. Third series, vol. ii. London, 1856, p. 428. Quoted fom British and Foreign Medico-Chirurgical Review, July, 1857.

of puerperal convulsions; in twenty-one of these cases the urine was examined, and with one exception, found to be albuminous. In the exceptional case, the convulsions depended on arachnitis.

In Dr. Bliss's four cases of convulsions, all had bloated countenances, and more or less œdema in other parts of the body. In one of these cases the urine was albuminous.*

Such, then, is the character of the evidence by which the connection between the presence cf *albuminuria* in the pregnant female and the production of *puerperal convulsions*, as the result of its toxæmic influences, is established.

The testimony is doubtless as full and conclusive as we could reasonably expect, when we remember how recently these views were first entertained, and the comparative infrequency of the manifestations of convulsions as a complication of gestation and parturition.

Amid the multitude of abnormal symptoms not unfrequently induced by the changes wrought in the animal economy by so important a physiological process as that of conception and the long subsequent period of gestation, not the least remarkable and interesting, is the disturbance caused in the healthy condition of the blood, and the addition of an element to the usual constituents of the renal secretion.

Albumen may be present in the urine of the pregnant female without the development of convulsions. Either the process of depravation may stop short of the point necessary to act upon the nervous system, or, the brain and spinal cord may not be impressible to the conditions of toxæmia present. An analogy may, perhaps, be found to the want of a response to the irritation of the toxicological condition of the blood, in ordinary tetanus. But very few of the innumerable number who daily receive punctured wounds suffer from a development of tetanic spasms.

In reference to the comparative frequency of the albuminous presence as a complication of pregnancy, and of the proportion of such albuminoid subjects in which convulsions follow as a sequence, it will, perhaps, be sufficient to quote the following authorities. Upon this point the figures are necessarily limited, as the opportunities for a regular and systematic examination of the urine of the pregnant female are unfrequent. It is only when the suspicion of positive disease is entertained that such opportunities are generally afforded. To ascertain how far such changes exist, and yet exhibit no sign of their presence, is a problem of difficult solution.

---

* Ante p. 713.

Dr. Blot* found albuminous urine present in 41 cases out of 205, primiparæ being chiefly affected.

But 7 cases of convulsions were developed in these 41 cases of albuminuria.

Dr. Litzmann examined the urine of 131 females: 79 during pregnancy, 80 during labor, and 80 after delivery. He found albumen present in 37, and absent in 95.

Of the 95 whose urine contained no albumen, 53 were primiparæ and 42 multiparæ.

Of the 37 who had albuminuria, 26 were primiparæ and 11 multiparæ; 2 were pregnant with twins.

Of the 37, the urine of 16 was found to be albuminous during pregnancy; in 10 of these the albumen continued some days after labor; in 4 it dis appeared before confinement.

In 4 women in whom albuminuria was found after labor, it had probably existed during pregnancy, although the urine had not been examined.

MM. Devilliers and Regnault report 12 cases of convulsions in 20 albuminuric subjects; and of and among these 20 cases, there were 11 deaths.†

In a recent paper upon this subject by M. Imbert–Gourbeyre, he gives 159 cases of albuminuria which have been observed in modern times, where there were 94 cases of eclampsia.‡

Positive organic renal disease is undoubtedly present in many of the cases, which may have existed previous to pregnancy, and been hastened to a full and fatal development by the conditions of gestation. Or irreparable injury may be inflicted upon these organs by the long continued congestion to which they are subjected. The unfavorable character of the prognosis in such cases is sufficiently evident.§

Happily, in a large proportion of the cases, the presence of albumen in the urine is not owing to such serious pathological changes. Renal conges-

* Amer. Journ. Med. Sciences, April 1853, p. 537.

† Med. Chirurg. Review, July, 1847.

‡ Brit. and For. Med. Chirurg. Review, Jan., 1858, p. 88.

§ Note. Prof. Simpson has published the account of three fatal cases of puerperal convulsions: one occurring during labor; the second, the first fit happened a month before delivery; the third, eclampsia supervened seven weeks after labor and the apparent perfect recovery of the patient. In two of these cases there was positive evidence of organic change; and in the third, a whitish turbid fluid was found in the renal pelvis, looking like pus. See American Journal Medical Sciences, January, 1848, p. 88.

tion simply, is regarded as sufficient for its production, induced most frequently by the pressure of the gravid uterus. The perfect recovery of the patient after delivery, is proof of the absence of serious organic changes in the kidneys, such as is generally known as Bright's disease.

Mechanical pressure, in which is included the resistance offered by the tense, unyielding abdominal walls of most primiparæ, is we believe, admitted by most writers upon the subject, to be sufficient to develop the presence of albuminuria. The tight girding of the abdomen often practiced as a measure of concealment, by those pregnant out of wedlock, has been adduced as an additional means of exerting injurious pressure. The fact of the artificial production of albuminuria by the ligation of the renal veins, is cited in evidence of the morbid results likely to follow from arrest of the due circulation of the blood.

The sufficiency of mechanical pressure in whatever way induced, to develop albuminuria, is admitted by Simpson, Lever, Cormack, W. Tyler Smith, and Litzmann.

Five of Litzmann's patients who had albuminuria, were pregnant with twins.

Dr. W. Tyler Smith has thus graphically described the results of pressure. He says, "It was necessary to take a comprehensive view of the causes of blood-poisoning dependent on the pressure of the gravid uterus. There was the pressure on the intestinal canal, causing constipation; there was the pressure upon the emulgent veins, causing albuminuria, and the retention of urea in the blood; there was the pressure upon the hepatic vessels, which he had frequently observed to produce pink deposits in the urine; and lastly, there was deficient oxygenation of the blood from pressure upon the thoracic viscera.*"

In a later paper upon the subject, Dr. Smith observes: "I have seen it occur in primiparous women of relaxed habits, as early as the fourth or fifth month, where the abdominal walls were flacid, and no apparent pressure or tension existed. The disease appears to me in such cases to depend upon sympathetic irritation of the kidneys by the gravid uterus, similar with the irritation of the salivary glands, the mammæ, thyroid, &c., and not upon mere pressure."†

We could scarcely expect the opinion as to the sufficiency of renal congestion for the prodnction of these several symptoms, to be accepted without

* Med. Gaz., June 15, 1849, as quoted in Braithw. Retrospect, Part xx, p. 198.
† Lancet, March 1, 1856, p. 221.

any dissenting voices, and we accordingly find this position attacked by such men as F. Wieger, P. Cazeaux, and Imbert–Gourbeyre, who all contend for positive organic changes as necessary to the production of eclamptic seizures; but it will not escape observation, that what constitutes the essential character of these organic changes is a question which they are not agreed upon among themselves.

Dr. F. Wieger,* maintains "that up to the tenth day of the puerperal state, anatomical lesions are always found; and that profound alterations of the kidneys are more frequent than congestive conditions. That the kidneys cannot pour out albumen in any considerable quantity, or during a certain time, without becoming clogged up and diseased."

Cazeaux† contends for the necessities of a Bright's disease to develop puerperal eclampsia, but he refines his etiology of the former disease so far, as to insist that Bright's disease does not primarily or mainly depend upon positive, or recognizable changes in the anatomical character of the kidneys, but that Bright's disease is primarily a change in the character of the blood, and that albumen may be excreted by the kidneys without appreciable changes in these organs.

M. Imbert–Gourbeyre‡ contends "that puerperal albuminuria is neither more or less than Bright's disease, (improperly called albuminous nephritis,) there being a puerperal Bright's disease just as much as there is a puerperal pneumonia or peritonitis. Moreover, puerperal eclampsia is nothing else than puerperal Bright's disease, in which convulsions occur; or in other words, the eclampsia is really sympathetic of Bright's disease."

The weight of opinion is, however, as we have previously stated, that this albuminoid condition is most frequently independent of any anatomical lesions, and results simply from renal congestion, and that the kidneys resume their normal functions in a few days after the removal of the disturbing causes by delivery.

Dr. Braun§ gives the alterations which the normal proportions of the constituents of the urine undergo in uræmic eclampsia, as follows:

"The uric acid is diminished.

"Urea diminished or almost wanting.

---

* British and Foreign Med.-Chirurg. Review, Oct. 1855, p. 27.

† Cazeaux's Midwifery. Sec. Albuminuria, p. 288.

‡ Prize Paper: Puerperal Albuminuria. Mémoirs de l'Academie de Médicine, 1856. Quotation from Brit. and For. Med.-Chirurg. Review, Jan., 1858, p. 86.

§ Brit. and For. Med.-Chirurg. Review, Jan., 1855, p. 42.

"Chlorides but little altered.

"Sulphates and phosphates either diminished or increased.

"Uroxanthin increased.

"The specific gravity of the urine varies from 1010 to 1030. If the sediment of the urine be examined in the first twenty-four hours, blood and mucous corpuscles and epithelial scales will be found, but these disappear when decomposition ensues. The more acute the disease of the kidney, the more cloudy the urine, and the greater the number of blood-corpuscles. The urine is generally acid, and gives its characteristic deposit with heat and nitric acid."

*Symptoms.* The period at which the renal affection commences in pregnancy, is unknown. It usually begins insidiously and increases slowly. The only constant sign by which renal disorder is denoted, is exhibited in the condition of the urine. Devilliers and Regnault have observed albumen in the urine at the sixth month; Litzmann not before the eighth month. Dr. W. Tyler Smith has seen it as early as the fourth or fifth month.

Cazeaux says he once detected it at four months in a greatly infiltrated primiparous female, who was delivered at six months of a still-born child, and whose urine was slightly albuminous eighteen months afterwards. M. Bach, of Strasbourgh, says that he has seen it at six weeks in a very nervous person. M. Cahen mentions three cases in the fifth and sixth months, and M. Bach two others.

The quantity of albumen is usually very conspicuous, and increases as the time of delivery approaches. In proportion to the intensity and duration of the morbid process in the kidneys, are found casts of the uriniferous tubes in greater or less quantity, the epithelium lining them being sometimes normal, sometimes in a state of fatty degeneration. In the milder cases, the tube-casts are often found just at delivery, or soon after.

Lumbar pain is frequently present, but not so constant as to be diagnostic. Dr. Litzmann has found tenderness on pressure over the kidneys in nearly all the cases—this condition not being observable in pregnant females whose kidneys are unaffected. The patient complains of headache, and sometimes of dimness of sight and of amaurosis, and there is general pyrexia.

Dropsy may be entirely absent, but it is generally present. When present, the development of the dropsy is sometimes rapid and acute, but is generally gradual. It may be limited to the lower extremities, but it usually extends to the upper extremities and face; the precipitate of albumen being as great in the one case as in the other. The œdema may vary from mere puffiness to excessive distension, and may invade the splanchnic cavities. In the progress of the disease to its fullest severity, the legs become excessively swollen, and the vulva and vagina so tumid as to render an examination difficult. There is puffiness of the face and hands, œdema of the abdominal walls, and almost every part of the body pits deeply on pressure. Œdema of the upper part of the body, the hands, arms and face is regarded as a most unequivocal sign of renal disease.

The amount of cerebral disturbance in some of the cases is very considerable, as manifested not only by headache, but by disturbance of vision, which may range from slight dimness of sight to complete amaurosis. The cerebro-spinal symptoms which precede the attacks of uræmic convulsions, bear the closest resemblance to those of Bright's disease. Upon the frequency of these symptoms, as well as such other general disturbances of the system, as he calls premonitory symptoms, Dr. Wieger* has collected the following figures: "In seventy-eight cases of Bright, Barlow, and Frerichs, there was amurosis and amblyopia ten times; syncope nineteen times; singing in the ears and deafness ten times; convulsions fourteen times. Out of one hundred and forty cases of eclampsia collected into a table, forty-three showed premonitory symptoms. Of the cases in which the eclampsia broke out before labor, there were forty per cent.; of those in which it began during labor, thirty per cent.; and of those in which it began after labor, twenty per cent., which were attended with premonitory symptoms. As premonitory symptoms, the author enumerates vomiting and diarrhœa, but principally headache, disturbance of intellect, and often delirium; cramps and amblyopia, sometimes followed by blindness, not unfrequently precedes. The changes of the pulse and pupils were too uncertain to be considered."

Notwithstanding the close connection of all these symptoms with a condition of albuminuria, and a uniformity of sequence so constant as to be well regarded in the order of cause and effects, Dr. Litzmann declares he has several times seen œdema of the extremities, including even the upper part of the body, the hands, arms and face, accompanied sometimes even by headache and transient disturbance of vision, when there has been no albumen in the urine.

The absence of albumen in these cases is, perhaps, no more remarkable than the two cases cited by M. Depaul, "in which, having examined the urine before labor without finding any albumen present, convulsions broke

---

* Brit. and For. Med.-Chir. Review, Oct., 1855, p. 427.

out; and the urine was found to contain albumen after the second fit in the first case, and after the fourth fit in the second case."

M. Imbert-Gourbeyre says, "at the present time, it is incontestable that puerperal eclampsia may exist without albuminuria."

Cazeaux admits the *bare possibility* of the development of convulsions without the presence of albuminuria; a form of expression intimating his doubts, after all, of its occurrence.

*Treatment.* The treatment of puerperal convulsions, the most readily accepted, and the form long prevalent, could be no other than that inculcated by the theories taught and accepted as to their cause. Fortified by a pathology which looks to a condition of congestion as the exciting, or at least, predisposing cause of these convulsive seizures, the treatment which almost as a matter of necessary sequence flowed from it, is one of depletion,—blood-letting and evacuents are the means to be employed as a necessary consequence of this view of the causes concerned in the production of these convulsions.

Gooch says, "Bleeding is our sheet-anchor, in *whatever class of patients* the disease may occur; and to be efficacious, it must be employed boldly and speedily, and to a greater extent than is generally imagined. * * * I can now conscientiously declare, that I never had a patient die of puerperal convulsions when the disease has been thus boldly treated; those who have died have been bled with a sparing hand, and to an insufficient amount."*

Prof. C. D. Meigs says, "It is scarcely worth while, almost, to open a vessel to draw off eight or twelve ounces of blood. The patient ought to lose from thirty to sixty ounces at one venesection, if possible; and if signs of faintness appear they should be hailed as the harbingers of success."†

But in reference to *treatment*, it is the more especial duty of your committee to collect the facts having reference to the employment of *chloroform* and the other *anæsthetic agents* among the remedial measures in the management of puerperal convulsions, and to estimate, as far as practical, their value either when associated with other means, or employed alone as curative agents.

No better method of accomplishing this object has occurred to your committee, than to collect as far as practical, the cases in which this treat-

---

* Practical Compendium of Midwifery. By the late Robert Gooch, M. D., p. 233. (The italics are our own, used by way of emphasis.—COMMITTEE.)

† Obstetrics, the Science and the Art. By C. D. Meigs, M. D., 1852, p. 458.

ment has been employed, as they are scattered through our medical literature, or as they may be communicated through the kindness of friends directly to the committee.

In no other way can the different phases of the disease, or the peculiar circumstances surrounding each case be so well exhibited. We only regret, that so many of the cases are so imperfectly reported as to detract much from their real value as means of instruction; and that the resources at our command are not such as to enable us to furnish as connected a series in the chain of evidence as we could wish.

It will be observed that but few of the cases are pure chloroform cases. In the greater part, chloroform, or ether has been employed only as an adjunct to other measures. In the majority of the cases, blood-letting has preceded the use of the anæsthetic agents, and the chloroform been employed rather as a secondary than a primary means of cure. In some of the instances, however, such has not been the case, but the anæsthetics have been employed for the direct purpose of quieting the nervous system, without the intervention of the lancet.

At the conclusion of these cases we shall endeavor to analyze them, and present in a connected order the lesions to be learned from their study.

We propose to present the cases as far as possible in their chronological order, as indicating the successive steps by which this mode of treatment has been introduced. This will account for the mingling of foreign and home reports.

Case I. W. J. Kite, Esq., of Hatfield,* relates a case of puerperal convulsions, in which chloroform seemed to exert a most beneficial effect.

The patient was thirty years of age, and in her second labor. After irregular pains had existed for about twenty-four hours, she was seized with convulsions; bleeding was employed, but the convulsions continued. In about thirteen hours more, the membranes having been ruptured for some time, the os dilated to the size of a crown piece, and the convulsions still recurring at short intervals, Mr. Thomas suggested the inhalation of chloroform, as the most likely means of affording relief. A sponge containing chloroform, was held to the nose, and its effects were soon visible. In less than five minutes she became perfectly quiet, and the convulsions ceased. In ten minutes, contractions of the uterus came on, and continued regularly; the head of the child gradually advanced, and as it descended into the vag-

---

* Braithwaite's Retrospect, Jan., 1848, No. xvii, p. 320.

ina, the hæmorrhage ceased. In one hour and a quarter from the first inhalation, the influence being kept up the whole time, she was delivered of a male child, and without having any recurrence of the convulsions. The inhalation was now discontinued. The placenta followed in a few minutes; the uterus contracted, and no hæmorrhage followed.

CASE II. Mr. Fearn, of Derby,* has also given chloroform with excellent effect, in a case of puerperal convulsions. The woman had been insensible during the whole day, with frequent convulsions, which ceased almost immediately after the chloroform took effect; and when completely under its influence, she was delivered with the crotchet.

CASE III. Mr. Clifton, of Welwyn,† relates another case in which it was equally efficacious. Notwithstanding the most violent convulsions, the woman sank into a tranquil sleep in a minute or two after its use.

CASE IV. *Reported by Samuel Cabot, M. D., of Boston, Mass.*‡

March 13, 1848. Mrs. H., strong and healthy, 27 years of age. Married at seventeen; mother of four children. Always has had very short and easy labors of about ten minutes duration. Supposed herself to be at about the full period of gestation. Fell upon her back with considerable violence,

---

* Braithwaite's Retrospect, Jan., 1848, No. xvii, p. 320.

† Ibid.

‡ A Treatise on Etherization in Child-Birth. By Walter Channing, M. D., Boston. 1848. Page 393. The Report is also found as Case LV, p. 251.

NOTE. So far as we have been able to learn from the reported cases which we have examined, this is the first instance in which anæsthetics were administered in puerperal convulsions in America; but so far as our knowledge extends, it was not given to the world until Dr. Channing published it in his " Treatise on Etherization in Child-Birth," which work was not issued until towards the end of the year 1848.

We believe the succeeding case of Prof. J. P. White, of this city, to be the *first published American case* of the use of anæsthetics in puerperal convulsions; and we know he suggested its use, and administered it without being aware that it had been previously employed in such cases by any person.

We make this reference actuated by our sense of local pride, which prompts us to secure for the members of the profession of our city every claim to which they are justly entitled; as well as from a desire to accord full justice to the claims of an individual member of the profession, for the introduction, or sanction of a remedial measure, promising to be of so much value in these cases.

about a week before and had had some nausea, with uneasiness in head, from that time.

At 8, P. M., March 13th, was found upon the bed in a violent convulsion, blood flowing in considerable quantity from mouth; convulsions recurring every fifteen minutes. Was seen by Dr. Cabot at 9 o'clock. The muscles of head, chest and arms principally affected, diaphragm also; left side rather more than right. Left pupil dilated. Head of child very high, and mouth of womb not felt. Took sixteen ounces of blood from arm. Ice to head and sinapisms to feet. Pulse strong and full before venesection; small and feeble after. In about thirty minutes after venesection, recovered senses for a short time, so as to answer questions coherently. Had another convulsion at an hour and a half after venesection. Bled her again at 11¼, to ℥xviij. After venesection, quite pale; pulse small and weak; pupils fixed, and rather contracted, but equally. In an hour, had another fit; and one each successive hour, for three hours; then becoming oftener, until once in twenty minutes.

At 3½ o'clock, A. M., 14th March, Dr. C. applied, at the apparent commencement of a convulsion, a sponge, saturated with *sulphuric ether*, over her mouth and nose. The spasmodic action soon ceased; and in about three minutes she was in a deep sleep. The head of child was somewhat advanced; could only reach anterior part of neck of womb; rather soft and much shortened. Ether was used at every threatening convulsion. Almost immediately after patient was obviously etherized, regular labor-pains commenced, and continued at short intervals, until at 6, A. M., the child was born alive. Ergot, grs. xx, was administered, as womb did not contract well.

After the birth of the child, patient slept quietly about an hour; then had another convulsion, there having been an interval of nearly four hours since last spasm. Ether was again used; but pulse became so feeble and small before any effect could be produced, that it was discontinued, and an enema of tinct. opii, ʒj, and pulv. asafœtid., ʒj, in half a cup of starch was administered; and hyd. submur., grs. xv, given by the mouth. After this, patient slept quietly for three hours, then had a convulsion every half hour, until 2, P. M.; when after considerable difficulty, got down tinct. opii, gtts. xxx, pulv. asafœt. grs. x. Had one more convulsion soon after taking it, and then slept quietly for two hours; and then beginning to move as if about to have a convulsion, gave her tr. opii and asafœt., as before; and after a few struggles not amounting to convulsions, became again quiet, and remained so until about half past five, when, after having taken some senna and salts, convulsions again came on: the first continuing about half an hour, and hardly ceasing before another commenced, which lasted about the same time; then a short interval, and then another not severe convulsion; after which, she had no return of them, and the case progressed favorably, with a perfect recovery. (*Condition of urine not noted.*)

CASE V. *Reported by Prof. James P. White,* Buffalo, N. Y., July 18th,* 1848.

Case occurred in the practice of Dr. Devening. The patient, an Irish woman of full habit, in her third labor; her two previous deliveries had been accompanied by no unusual symptoms. Prof. White in consultation.

The convulsive paroxysms succeeded each other with great rapidity; the face flushed and the pulse full. Full *bleeding* was performed twice, but the convulsions continued with but slight diminution.

Prof. W. advised and administered chloroform. The muscular agitation, which before could not be controlled, now subsided, and delivery of a pair of twins was effected without difficulty; both children living and doing well. The recovery of the patient was rapid and complete. (*Condition of urine not noted.*)

Dr. Walter Channing, in his work upon Etherization in Child-Birth, has given the particulars of 79 cases in which anæsthetics were administered, during the different stages and in the different complications of labor. Eight of these cases were *puerperal convulsions.*

As Dr. Channing has been not only an ardent advocate of the safety and propriety of the administration of anæsthetic agents in obstetrical practice, as well as the diligent student in collecting such facts as would serve to sanction its use from the experience of the profession, we deem no excuse necessary to refer to such evidence as he has furnished us of its safety and utility in modifying and controlling the convulsive seizures of the pregnant and parturient female.

We shall condense these cases as much as possible and give their principal points of interest only.†

---

* Buffalo Medical Journal, vol. iv, Sept., 1848, p. 206.

† NOTE. If any apology to the Association, for occupying its time with the recitation of cases which are already made accessible in book-form to the profession, be necessary, we reply, we know of no other way of preserving and presenting the historical order in which these agents have been made to assume a rank among the remedial measures applicable to the treatment of puerperal convulsions. Besides,

CASE VI. (Case 57, page 254.) Aged thirty-three; three children at full time; one at seven months, accompanied by profuse hæmorrhage, and three abortions. Is now seven months pregnant. Has had poor health during the whole time. Has gradually become *universally* and *greatly anasarcous.*

On the 9th, severe headache; took but very little food. Headache increased, P. M.; and at about 1, A. M., of 10th, was seized with convulsions. Got active enemata soon, and inhaled sulphuric ether. Great excitement followed, becoming almost uncontrollable. Chloroform was substituted for ether, and the change in things was almost immediate. Great quiet took place; and though fits continued, their intervals were long, and she was partially conscious during them. Headache continued.

Attempts had been made to bleed, but failed. Leeches applied to temple. Chloroform continued.

Dr. C. saw the patient between 4 and 5, A. M. The convulsions recurred at long intervals, with violence. Some consciousness in the intervals. Os uteri closed, very high up.

About twenty ounces of blood were drawn from a large orifice. The leech bites continued to discharge blood quite freely. Chloroform was in use at the time; and after an inhalation, the bleeding ceased entirely, and did not recur. Convulsions continued as before; and it was now agreed to employ antispasmodic and narcotic remedies; the tinct. opii, and emulsion of asafœt., one drachm to an enema, and from thirty to forty drops of the former. These obviously, for a time, checked the convulsions; and at length, about 10, P. M., after various experience of their use, the fits ceased entirely and did not recur. She had a perfectly quiet night, and on the morning of April 11, was comfortable and conscious. On the day before sub. mur. hydrg., grs. xv, had been taken. *Renal secretions natural.*

12th. Bowels moved sufficiently. Night comfortable. Child born dead at 12 to-day, at noon. Recovery perfect.

CASE VII. (Case 1, page 261.) May 7th, about 4, P. M., was seized

---

as we have already shown, so firmly fixed is the congestive pathology, and depletory treatment of this disease, in the minds of the profession, no damage can be done in accumulating evidence upon a point, which if sustained by testimony and experience, promises to introduce a less sanguinary, and no less successful form of treatment.

with most severe pain or cramp in the stomach, without any error in diet, or from any known disease, or change from ordinary health. In the evening, convulsions. These were very severe. They had been preceded by that state of stomach to which Denman and others ascribe such important agency in the production of this most formidable disease. For a time, some consciousness occurred between the fits. This was not of long continuance. Blood-letting, and leeching to head, were practiced. But the skin was pale and cold, and the pulse feeble; and much blood was not taken,—and this with very little, if any, relief.

Chloroform was inhaled, but did not diminish the violence, or increase the intervals between the fits. These, for the most part, were about half an hour; once, an hour and a-half. *Urine*, drawn by the catheter, showed some striking deviations from health. It was of a blackish-brown color, not at all resembling urine; and in smell still more unlike it, being entirely destitute of urinous odor.

First seen by Dr. C. between 12 and 1, noon, of the 8th. She was perfectly unconscious; breathing heavy and noisy; pulse very rapid, small, wiry; much heat especially about the face. The face was swollen, leaden, pale, and haggard. *Anasarca* existed everywhere; her lower extremities were especially swollen. Labor had begun. Os uteri dilated somewhat, and dilatable.

A violent fit soon came on. During the height of the fit, the pulse at the wrist was wanting. As chloroform had not controlled the fits, sulphuric ether was inhaled. For a short time, the intervals were lengthened; but after this, the fits became as frequent, and more so than before. Labor had advanced, but the state of the patient was obviously growing worse. The disease had now existed many hours and was obviously increasing. The membranes were punctured. Some temporary diminution of the fits followed, but soon became as pronounced as ever.

The child was delivered by instruments, and during the operation and for some time after, fits did not occur; the pulse improved, was slower, and gained strength; breathing was good, and lividity entirely disappeared.

Convulsions recurred soon after, but ceased again. She sank gradually and death took place forty-eight hours from the birth of the child.

Case VIII. (Case 69, page 278.) Mrs. ——, aged eighteen; first labor. Was taken with distinct uterine contractions, July 21st, at about 11, P. M. Had slept badly, and had irregular and painful uterine action for three or four preceding days. Labor had advanced very favorably, though with much complaint, till 9, A. M., of 22d; when without any observed precursors, except very slight headache, convulsions occurred. In the intervals between the first four fits, consciousness returned. The jaw was dislocated during one of these attacks, and the difficulty was pointed out by the patient. Reduction was at once accomplished, but partial dislocation occurred twice afterwards. After the fourth convulsion, consciousness did not return. The labor made steady progress, and at noon, a boy of large size and alive, was born. The labor was completed during the sixth convulsion.

Extreme restlessness accompanied and followed the contraction which terminated the labor, lasting an hour. This violent action ceased as the next fit manifested itself; and from this time, there was alternately a fit, and an attack of excessive restlessness, similar to the first above described.

The treatment consisted in blood-letting, a solution of tart. antimon., iced water to the head, and leeches to the temples.

The convulsions continued, and increased in violence and in frequency; and about 6, P. M., Dr. C. was called in consultation.

The pulse was rapid, between 130 and 140, and not deficient in strength. The skin was very hot, and perfectly dry. The face was generally pale, the lips being quite red. The eyelids were naturally closed, and a state of rest or of quiet sleep which Dr. C. had rarely observed in genuine puerperal convulsions. Unconsciousness entire. The abdomen was full, partly owing to flatus; but a portion was produced by the womb, which was very large, extending above the umbilicus, and very hard. Urine has been passed during the day. A fit soon came on; accompanied by distinct uterine contractions, with evidences of pain in that region.

Ten grains hyd. submur. were given, and the administration of sulphuric ether commenced. The ether was renewed three times in the course of an hour. There was unconsciousness before inhaling it. The principal change observed was the greater quiet, and the increased length of the interval between the fits after inhalation.

She had a fit in about an hour after etherization. The pulse was now more frequent, smaller, and the convulsion very severe. Asafœtida, ʒj. and tinct. opii, gtts. xxx, in an emulsion, by enema, was now given and retained. Quiet was now decidedly greater after the enema. Gentle sleep, with very easy breathing, came on. The complexion and expression were more natural. The last convulsion was at five minutes before 7, P. M. Another injection of asafœt. et tinct. opii, was given at about 10. The catheter was introduced and much *dark colored urine* drawn off.

23d. Night tranquil; no convulsions; takes drinks readily. At visit between 5 and 6, A. M., pulse from 96 to 100, of good strength. When called by name, answered, "What?" Flatus has been freely discharged, but no dejection. An enema, followed by ol. ricin., if required, was directed. A pint of urine, similar in appearance to that before drawn off, was removed by catheter.

24th, 8, A. M. Report of day and night favorable. Five dejections. Pulse 108, soft, sufficiently full. Countenance and manner natural; takes food with relish; semi-consciousness. Answered a question, though not disposed to do more. Catheter.

25th. Better.—Recovery perfect.

Case IX. (Case 71, page 285.) Aug. —. Aged twenty-seven; first labor; seventh month. Anasarcous to an unusual extent; lower limbs especially, enormously swollen. Has lately had paroxysms of severe distress in the epigastrium. Eat indigestible food for supper, and woke in the night with acute pain in the stomach, and took laudanum for same, with relief. Very soon after, was seized with severe pain in the head. Violent convulsions soon occurred. Imperfect consciousness continued a short time after the attack.

Was bled about forty ounces from a large orifice. The pulse became small, weak; face pale; respiration feeble: the whole state showing approaching syncope. There was very little, if any, change in the fits; an hour for the most part recurring between them; but not the slightest consciousness manifested itself in the intervals. Enemata, laxative and quieting, were given,—cold, assiduously applied to the head,—attempts made to get medicine into the stomach.

Labor at length occurred, and the membranes gave way.

At about 6, P. M., visited by Dr. C. Found her in apparently easy sleep; no stertor nor apparent trouble in respiratory apparatus or function. The complexion was clear, and no lividity anywhere noticed. The os uteri could be barely reached; it admitted the finger and moderately dilatable. Supposed labor to be present, and that it would be accomplished by nature, or art easily. As nothing was to be done immediately, left the patient to the care of her physician.

Dr. C. learned that after other means had been faithfully tried, and without the least apparent benefit, that sulphuric ether had been inhaled. At first, inhalation was attempted *during the convulsions;* but it was in vain to use it at this time, for ordinary respiration was then almost impossible. Inhalation was next tried in the intervals of the fits. The change which followed was in the length and the severity of the convulsions, these becoming less; and from 6, P. M., she had no more fits. Labor now advanced; and about 8, P. M., was terminated by instrumental aid. No improvement followed; and about 5, A. M., the next morning, death took place.

Reference is made to the attendant circumstances of the universal and excessive anasarca and the violent pain of the stomach, preceding the attack, by the reporter.

Case X. (Case 77, page 287.) Age twenty; first pregnancy, end of ninth month.

Aug. 12th, about 11, A. M., called in consultation. Learned that Wednesday night, 9th, was passed without sleep, and great restlessness and without apparent cause. Thursday made no complaint. Eat much food, which was strongly craved. In night was seized with intense headache, most severe in forehead, and represented by patient to be insupportable. At 2, vomiting occurred. Matters vomited, were large quantities of bile, of dense mucus, and portions of food perfectly undigested. The night had, like the preceding one, been passed without sleep, and in continuous suffering.

Between 8 and 9, A. M., of the 12th, was visited by her physician, and was seized in his presence with a convulsion. It was very violent, exactly resembling epilepsy, producing great and general agitation and livid face and hands. Heavy coma, with deep apoplectic stertor, followed. She had been in perfect health during the whole of pregnancy, and become fleshy and plethoric.

She was immediately bled, and no improvement following, at 11, A. M., Dr. C. was called. A fit had just ended. Heavy stertor; respiration much embarrassed; skin hot, dry; face swollen, flushed; pupils contracted to a point; head hot; pulse rapid, but strong and hard. Dilated and very dilatable os uteri; labor present. Bled about twenty-four ounces more.

Relief followed venesection. Pulse, breathing, and general state of the patient, showed improvement. The skin became cool; flush subsided; pulse was slower; and perspiration was present. Labor was progressing.

Sulphuric ether was now inhaled with a view to prevent recurrence of fits. It was not well breathed, and it rarely is in the intervals between genuine puerperal convulsions. Respiration is always more or less disturbed at such times, or, if not obviously thus, is often rendered so by any external cause of embarrassment. Inhalation was cautiously continued for half an hour or more. The pulse became slower, being 120 instead of 144, the number after last convulsion.

A fit at 1, P. M., an hour and a-half from the last. This was comparatively slight, and was succeeded by less heavy coma and stertor, and a less morbid expression of countenance. The interval was half an hour longer than before etherization.

It was agreed to omit inhalation; to observe the progress of the case; and to aid delivery give an infusion of secale. The labor advanced very favorably. Increasing consciousness of uterine contractions, and some of the ordinary expressions of the pain of labor. And at length, the patient so far recovered perception, as to recognize her mother, and to call her name. Had not met with a better promise in any case of the kind, nor seen any more severe.

This favorable state and gradual improvement had now continued for three hours, it being 4, P. M. A fit very suddenly came on, just at this time. It was the most severe one which had occurred. It lasted longer, and in its violence threatened immediate extinction of life.

It was agreed to remove the child, which was accordingly done; and it was still-born.

Very little improvement followed delivery. Respiration continued embarrassed, and very slight return of conciousness was noticed. Injections of asafœtida were given; a sinapism applied to the epigastrium, and skin cooled by use of cold water.

At 7, P. M., a slight fit. Tinct. opii was now added to the injection. More quiet.

At 1, A. M., another but very slight convulsion, and this was the last. There was, however, no amendment in the following interval of disease; and at 1, P. M., of the next day, she died.

Case XI. (Case 20, page 198.) Sept. 13th. Mrs. —— was taken with pain in abdomen, resembling labor pain, but more like colic. Had severe cholera morbus in night. Next morning took castor oil and an injection, and was thoroughly purged. At 12, noon, was taken with convulsions.

This is her third pregnancy, period seventh month. In her first, she had convulsions at the seventh month, and was delivered by natural effort. In the second, at about eight months, had convulsions, and was delivered by the womb. Her physician, finding she had suffered before fits with severe headache, and was deeply red in the face, bled her largely. Her fits, however, continued very strong, not at all diminished by bleeding and previous purging.

Dr. C. saw her about 6, P. M. She was in a very excited state, talking constantly, and tossing herself about vehemently. Her face and lips deadly pale; skin cold; pulse rapid, 150 in a minute. Every thing showed a state of extreme exhaustion, still with remaining power to make great *muscular* exertions. No pains; slight dilitation of os uteri, but soft and dilatable.

She soon had a fit, a violent convulsion, followed by deep stertor and stupor. She came out of this in about seven minutes, and began to talk, and to throw herself about. This state continued. The membranes were ruptured; the womb did not act. The intervals of convulsions lessened. The pulse sunk. She lost power to speak or move, and lay wholly unconscious on her bed. It seemed impossible that she should long survive this state of things, and it was decided that an attempt should be made to deliver. The head was perforated, and after a long trial, the fœtus was removed. She had two convulsions during the operation.

Left her quiet at 2, A. M. of the 14th.

14th. Fits occurred after Dr. C. left. In the morning was very much exhausted. Pulse very rapid. Great restlessness. Apparently threatened convulsions. *Ether* was *now* for the *first* time employed. She soon became calm. Had two slight fits afterwards; but in the course of two or three hours fell asleep, and had a long and quiet repose. No more fits; but as they were again threatened, inhalation was employed, and with the best effects. At 7, P. M., was sleeping very quietly; pulse 112, soft, and of good strength. Skin warm and soft. Expression and complexion natural. *No urine.*

15th. Report favorable this morning. Urine, during operation of an enema, very copious.

Case progressed from this time, and recovery was perfect.

Case XII. (Case 79, page 390.) The patient was aged twenty-one years; and six months pregnant.

Visited Sept. 14th; previously attended by homœopathic physician. This patient had, for some time, occasionally suffered from various affections of the head, such as dizziness, sense of fullness, pain, occurring suddenly from emotions, exertion, &c. There was often flushing of face, rash on the neck and cheek, coming and going suddenly. On the 11th instant, pain in the head, referred to the top, had been unusually severe. On the 12th, more severe. Cold water was prescribed to head. Much anasarca, with œdematous fullness of face and neck, had occurred. Had a convulsion on the 13th, at 8, P. M. Consciousness occurred after the spasm ceased. After about an hour's interval, another spasm; and two more followed, consciousness still continuing.

Opium was given in *homœopathic* doses, and four hours interval followed. Spasms then recurred. Opium now failed to check them, and veratrine and belladonna were given, after the Hahnemann plan. After 7, A. M., 14th, and when a number of convulsions had occurred, consciousness was abolished. Homœopathy was then abandoned. About ℥xx, of blood were next taken from the arm, and with some relief.

Dr. Channing was called at noon. Found the patient with a pulse of 120, not strong or full. Comatose; incapable of being roused. Skin hot, turgid, mottled; mouth open; tongue swollen, and protruded between the teeth. Os uteri and neck in ordinary state, at the supposed period of pregnancy. Uterine contractions had occurred.

A *pain* soon came on, and with it, a convulsion. The spasm was short, but little exceeding a minute; but as violent as Dr. C. thinks he ever saw. Heavy, almost suffocating stertor came on; and after some time, respiration became easier.

It was now agreed to use chloroform at the beginning and through the next *pain*, and to observe what followed. The spasm was over about ten minutes past 1, P. M. Contractions soon occurred, and chloroform was at once inhaled. No spasm attended or followed. *Pains* continued to occur, but no fits. At 4, another but lighter attack. It was sooner over, and the stertor less. The chloroform was now inhaled during a number of *pains;* then intermitted for one or more. Sinapisms were in the meantime applied to the lower extremities; a vesicating plaster to back of neck; cold to the head.

From 4 to 11, P. M., there was no fit. Contractions increased and os dilated. Chloroform was steadily continued. Once, *pain* being apparently less severe, a drachm of pulv. secal. was given as an enema. Good progress was made in the case.

Between 9 and 10, A. M., 15th, the child was born, and in an hour after was left with a good pulse and skin, and good promise of recovery, which promise seems to have been realized.

Case XIII. In connection with the above case, Dr. Channing relates briefly a case detailed to him on the day succeeding the above occurrence, by a professional friend. In which very severe convulsions occurred, about thirteen hours *after* delivery. She had inhaled chloroform during labor. It was inhaled *during* the convulsions; and they were entirely controlled by etherization. They amounted to four in all. Great prostration followed the suspension of the spasm. Fair reäction occurred after about twelve hours. Gradual sinking and death, a fortnight from the termination of labor. After death, was found evidences of pneumonia in a portion of the middle, and lower part of upper right lobes.

The urine *coagulated* by heat, confirmatory of Prof. Simpson's views; the kidneys were found normal; heart and brain healthy.

The two following cases have occurred later in Dr. Channing's practice and are copied condensed from the periodical in which they were first published:

CASE XIV. *Reported by Dr. Walter Channing,* Boston, Mass., Dec. 17th,* 1848.

Case a primipara, aged 22 years. A fortnight previous to the date of labor, (Dec. 17th,) was seized with convulsions; becoming comatose; breathing stertorous; pulse very rapid and small, and blood coming from nose and mouth; skin generally pale. A vein was opened, and thirty-two ounces of blood taken, followed by a perfect calm, and no return of the fits. In three or four days she walked abroad quite well.

A week after her recovery her physician called to see her. He found her complaining of most severe headache; referred to top and front of head, and almost immediately she said, "I can't see you, what is the matter?" He bound up her arm at once, and bled her again to full two pints. There were no convulsions.

On the 17th, between 8 and 9, A. M., labor had commenced. Her physician visited her very soon after, "in time to witness the faint fit which ushered in the labor." This was very severe; so that notwithstanding the former bleedings, and the pale skin, and very small and weak pulse, he thought it safer to bleed again, especially as such good effects had followed the former bleedings. He now took a pint. Convulsions continued to recur about once an hour through the day, the labor slowly advancing. At length, in the evening, progress had nearly or quite ceased; and the convulsions continuing, Dr. Channing was called in consultation.

Dr. C. found "the patient exceeding restles—very pale, lips as well as face,—skin cool, almost cold—pulse very small and feeble, about 140 in a

---

* American Journal Medical Sciences, April, 1849, p. 345.

minute. During pain, restlessness especially great. In intervals she lay most of the time in heavy sleep."

Dr. C. advised chloroform, which was used at once, being given in one of the intermissions between the convulsions, and she was speedily brought under its influence. Pains came on regularly, and efficiently; labor progressed rapidly; and delivery was speedily accomplished naturally. The womb contracted well, expelling the placenta, and was followed by no hæmorrhage. Dr. C. left the patient perfectly comfortable in about an hour and a-half after reaching the house.

The patient was seized with another convulsion about an hour after his withdrawal. Chloroform was at once used, and the fit was not as long nor as severe as had been others. There was frequent threatenings of fits during the night, by a convulsive twitching, or *drawing* of the head to one side, with rapid motion of the eyelids, the precursor of former convulsions. As soon as these signs manifested themselves, chloroform was inhaled, and not another fit occurred.

On the 19th, the patient was very comfortable; wide awake; color of cheeks good; pulse 96; warmth sufficient; says she felt "first rate." About a quart of urine was drawn off by the catheter, which resembled *turbid black tea*, into which much milk had been poured. It was entirely without smell, and had no appearance of urine. The application of heat, and nitric acid, gave most copious deposits of *albumen*.

Case XV. *Dr. Channing.**

"—— ——, aged fifteen; first labor; April 16th, 1849, at about 1, A. M. Began with vomiting. Physician found her in violent convulsions; was very fleshy, and in perfect health; no error of diet; full blood-letting, about ℥xxx; leeches to temple; terebinthinate enemata, cold to head.

I was called to see her about 1, P. M. Found her very ill; face pale; livid; respiration heavy, laborious; pulse from 192 to 200 in the minute, small, sharp; convulsions violent; os uteri dilatable; slightly dilated; show; head presenting. I advised chloroform; it was at once inhaled, and this very thoroughly; it checked the convulsions, so that no more occurred. The labor proceeded. At about 3, P. M., she recognized her mother, and gave other evidence of consciousness. I left her soon after, doing perfectly well. This patient was carefully watched, and whenever a convulsion was

* American Journal Medical Sciences, July, 1849, p. 43.

threatened, which was denoted always by wide-staring, fixed eyes, chloroform was immediately inhaled. In this instance, consciousness returned sooner than in any case which has come before me."

Case XVI. *Dr. Keith, in the Monthly Journal of Medicine*, August, 1850,* gives the following particulars of a case of puerperal convulsions treated by chloroform:

A lady, about the end of the seventh month of her first pregnancy, was suddenly seized with a convulsive fit, after suffering more or less for some days from headache, and slight feverish symptoms. The first fit occurred three hours before Dr. K. saw her, and in the interval she had three others. She had been bled, and the bowels had been freely opened. The fits ceased for a time, but after four hours they again recurred, and four followed each other in the course of less than an hour without any return to sensibility during the intervals. The bleeding was repeated very copiously, but seemed to have no effect in stopping the fits, or even in rendering them more moderate. Just as the fourth fit was coming on, chloroform was administered. It had an immediate effect in restraining the convulsive efforts, and she soon became perfectly quiet. Now, while under the influence of the chloroform, uterine contractions came on. The action of the chloroform was kept up, the labor went on steadily, and in six hours she was delivered of a still-born fœtus, without any recurrence of the convulsions. She slept for nearly an hour after the termination of the labor, when she awoke in a confused state, but perfectly rational and almost free from headache. She made a most favorable recovery; indeed she had not a single bad symptom from the time the chloroform was administered.

The urine was highly *albuminous*, becoming almost solid on exposure to heat. The coagulability gradually went off, and after a fortnight there was scarcely a trace of it.

Case XVII. At a meeting of the Newcastle and Gateshead Pathological Society, Mr. Andrew Bolton read notes of the following case:†

Elizabeth ——, aged 22, unmarried, at the full period of a first pregnancy, healthy, but for some time in a desponding way, was seized, Jan. 9th, at 4, A. M., with pain in head and loss of vision. At 8, A. M., I was summoned in time to witness a severe convulsive paroxysm, attended with stertor, livid-

* Quoted from Amer. Journ. Med. Science, Oct., 1850, p. 532.

† Braithwaite's Retrospect, Part xxix, p. 284.

ity of countenance, and apparent impending suffocation. Twenty ounces of blood were drawn from the arms, and the could douche unsparingly used to head and shoulders. In ten minutes she was calm again, the pulse reduced in tone 100. On examining the os uteri, it was felt high, slightly dilated, and extremely rigid. The mere introduction of digits sufficed to bring on the convulsions, which recurred again and again, with intervals of five or ten minutes, the whole muscular system participating in the spasms.

11, A. M. Paroxysms continue, and she is with much difficulty restrained. Two drachms of sol. morph. Ph. L. were given, producing an hour's repose.

12½ o'clock. Dose of morphia repeated, convulsions having returned with increased violence; countenance and general surface pale; the extremities cold. As her condition appeared hopeless, should the paroxysms continue, chloroform was administered on a piece of linen, in half-drachm doses, and its full effects kept up for three hours.

At 2, P. M., there was a slight return of convulsions; skin warm and perspiring; the os uteri was found steadily dilating; and from her uneasy movements it was apparent that uterine action had begun.

3½, P. M. The membranes were ruptured; and brisk uterine action ensuing, a dead child was expelled, immediately followed by the placenta. She regained her senses during the expulsive efforts, but appeared entirely ignorant of her previous condition. Recovery followed without any bad symptoms.

In conclusion, I would remark, that the convulsions were in no measure mitigated by the depletion, which was carried to the utmost; nor was there any yielding of the os uteri until the chloroform was inhaled.

Cases XVIII–XX. At a meeting of the New York Medical and Surgical Society,* Dr. Van Buren reported a case of puerperal convulsions, occurring in a lady, twenty-five years of age, with her first child. During gestation, she had enjoyed her ordinary health, except on the evening preceding the attack, when she had retired with a headache. Her physician was sent for at 1½ o'clock, A. M., and found her in a convulsion. Labor had not commenced.

An hour later Dr. Van B. was called, and found her in her third convulsion. It was now ascertained that the os uteri was beginning to dilate. Chloroform was immediately given, and after a time the convulsions were

---

* New York Medical Times, April, 1853.

relieved, when it was "let up," and the convulsions again returned. Its use was again resumed, and she was kept under its influence until about 6 o'clock, A. M., at which time the os being sufficiently dilated, the forceps were applied, and the delivery speedily completed. The patient was then left in a comfortable condition. Another convulsion, however, came on shortly afterwards, and they recurred every twenty minutes, except when chloroform was given, which controlled them. Other appropriate remedies were resorted to, but the anæsthetic was mainly relied upon. Its use was was continued until a few hours before death, which occurred fifty-two hours after the first seizure. During all this time there was *no secretion of urine.*

At the same meeting, Dr. Metcalf stated, that in half a dozen cases occurring in his own practice, and another half dozen in the practice of his friends, when chloroform had been relied upon, but *one case* had terminated fatally.*

Case XIX. Dr. McCready said, he had seen one case in which it was administered freely, but did not control the convulsions. This case, however, terminated fatally.

Case XX. Dr. Delafield narrated a case in which it was given without at all controlling the disease, and the result was fatal. In the only case which terminated fatally in his practice the last year, chloroform was used.

(Note. Dr. Metcalf also stated, that a case of his own, in which the patient had but one convulsion, terminated fatally. A *post-mortem* examination revealed a clot in the brain. The report does not indicate whether it was the fatal *chloroform case* referred to above, or not. This fact, an important one, is not mentioned.

Dr. M. thought "that the tendency to a recurrence of the convulsions is owing to excessive nervous excitability; and as chloroform will undoubtedly allay this condition, he thought its use highly beneficial.")

Case XXI. *Reported by Prof. James P. White, Buffalo, N. Y.*,†

*July*, 1853. Mrs. J., aged 24 years; third pregnancy; seventh month. After having suffered severely with what she supposed to be nervous

---

* This statement is so loose and indefinite, that the cases are not included in our analysis. It is simply given *for what it is worth* as a matter of testimony *in favor* of chloroform.

† Communicated to your committee.

headache, for two or three days, was attacked at about 10, A. M., with puerperal convulsions. Drs. Trowbridge and Wallis were called in and bled her moderately.

Prof. White visited her shortly after and commenced the administration of chloroform, and kept her moderately under its influence until 4 o'clock, P. M., without any recurrence of the convulsions. The chloroform was now suspended and the convulsions returned.

The mouth of the uterus being now dilatable, Prof. Flint and Dr. Wallis being present and concurring, and administering the chloroform, Dr. White turned and delivered. There was no return of the convulsions, but the patient was kept moderately under its influence for some hours. The urine was *albuminous.*

Recovery was perfect, and the lady has since born two children without any recurrence of convulsions.

Case XXII. Dr. Hecker* relates the following: A primipara, æt. 23, had been in good health up to the time of labor. On the evening of the 6th December, she took a rich supper; she was taken ill in the night, and fell into convulsions. A period of four or six weeks was yet wanting to the term of gestation. No œdema.

The legs exhibited a perfect tetanic rigidity, and she foamed at the mouth and gnashed her teeth. Immediately on the cessation of a fit some urine was drawn by a catheter. The secretion was acid, and an enormous quantity of *albumen* was thrown down by heat, Chloroform was exhibited, and the uterine douche employed to hasten parturition. The douche had the desired effect; but the chloroform did not in the slightest degree diminish the intensity or frequency of the fits. The patient died soon after delivery.

An extravasation of blood, the size of a dollar, was found under the arachnoid, on the left hemisphere; and a similar one, but smaller, on the right hemisphere. The left kidney was shrunk to the third of its normal size, weighing only two ounces, and quite unfit for the excretion of urea. The right kidney was normal.

Case XXIII. *Reported by Prof. James P. White, Buffalo, N. Y.*†

1855, *Feb.* 13*th.* Mrs. ——, aged 27 years; second pregnancy. Labor

---

* Brit. and For. Med.-Chirurg. Review, July, 1854, p. 205.

† Communicated to your committee.

anticipated about a fortnight. Somewhat dropsical, extremities œdematous, but urine had not been tested.

During the night of the 12th and 13th, and morning of the 13th, she had had five convulsions previous to, and six convulsions succeeding to delivery; and had been freely bled.

At 11, A. M., was visited by Dr. White in consultation. Convulsions were occurring at this time as often as once in an hour, and were very severe. Coma profound. Pulse 160.

The administration of chloroform was immediately commenced, and continued until seven or eight o'clock at evening without any further return of convulsions. Upon the withdrawal an opiate injection was given.

Convalescence was protracted, but recovery was finally perfect.

The urine was tested during the illness and found to be *albuminous.*

This patient has since given birth to a healthy child at full period without any recurrence of convulsions.

Case XXIV. Dr. Hecker* has also given the following case, evidently for the purpose of establishing the value of the means used for the purpose of inducing premature labor, but as *chloroform* was employed in the treatment, and as the short abstract of the report contains *contradictory* testimony as to its influence, we give it for what it is worth toward establishing its value as a remedial agent. The active *mechanical means* resorted to in the case, undoubtedly should not be lost sight of in the estimate:

"A strongly-built primipara, aged 20, fell into convulsions, with loss of consciousness, in her sixth month; the urine contained a great quantity of *albumen;* the legs and abdomen were œdematous. Cold affusions to head, mustard cataplasms, *chloroform, were useless.* To bring on labor, India-rubber bladders were applied to the breasts, and at the same time, the colpeurynter to the uterus; besides this, the os uteri was dilated by the finger. The most *violent convulsions* during the operation, *were moderated by chloroform.* The child breathed some hours. Consciousness returned on the birth of the child. The albuminous character of the urine lasted, gradually lessening, till the fourteenth day. The woman recovered slowly."

Cases XXV–XXVII. M. Frémineau,† Feb., 1856, report three cases, in

* Brit. and For. Med.-Chirurg. Review, April, 1855, p. 440.

† Brit. and For. Med.-Chirurg. Review, July, 1856, p. 211.

which chloroform was employed, of which the following brief and imperfect abstracts have been given to the English reader:

1. A primiparia, aged 25, at end of gestation had general cephalgia, redness of face, vertigo, vomitings. At access of labor a sudden intense fit of eclampsia, with protrusion of the tongue, which cut and compressed by the teeth, became so swollen that asphyxia seemed imminent. This state lasted for two days. Delivered of a living child. Symptoms persisted; convulsive fits very frequent. Chloroform inhalations, lasting twenty minutes each, and an injection of chloroform. After each inhalation, the patient fell into a state of complete sedation; then, when a fit appeared imminent, she was again submitted to anæsthesia. In the evening she was much better. This treatment was continued for three days, during which time the fits gradually diminished in frequency and severity.

Case XXVI.—2. A primipara, aged 24, six months pregnant, had been seized two days with eclampsia when admitted, (25th March, 1855,) into the Hôtel-Dieu, under M. Piedagnel. Loss of consciousness, dilated pupils, convulsive movements, *no albuminuria.* Attacks followed by hemiplegia. Venesection, and potion containing twenty minims of chloroform. Next day, another attack; same treatment. 27th, another attack; venesection. From the 1st to the 7th April, the consciousness and movement returned; at times, still stiffness in limbs; prolonged baths. The 11th, a violent fit; venesection, and chloroform potion. Patient improved. The 14th, after some annoyance, another violent fit; venesection, chloroform potion. Patient now very anæmic. Question of artificial delivery discussed; postponed. Slight convulsive attacks recurred at intervals until delivery on 13th May. Child living. No attacks after delivery.

Case XXVII.—3. A primipara, aged 24, was delivered at the Hôtel-Dieu, in the night of the 21st–22d Nov. For some days before, headaches, vertigo; a fit of eclampsia on the 19th, the day when indications of commencing labor appeared. 22d, profound coma, dilated immovable pupils, stertorous breathing, and occasional convulsive shocks; chloroform potion. 23d, better. 24th, coma, but no convulsions. 25th, consciousness. From this time gradual recovery.

(Note. The value of the above threecases is much impaired by the imperfect manner in which they are reported. Case 1 is not full, and 2 and 3 are especially imperfect. An entire month is passed over in Case 2, without an account of treatment; and what influence the chloroform *potion* had ascribed to it, is not mentioned. Whether any other treatment in Case 3, besides the chloroform potion, was employed, is not recorded.)

CASE XXVIII. *The following Case has been furnished the Committee, by Dr. Strong, of Westfield, Chautauque Co., N. Y.*

*July* 31st, 1856. Mrs. G., aged 22 years; first pregnancy; in the sixth month of gestation. Saw her in consultation with Dr. Pupikofer.

Had exercised too much the day before, and to add to the trouble, had slipped down, jarring herself. When I saw her, was having pains hourly, going into convulsions at outset of pain, and remaining in them about half an hour, followed by half an hour's quiet. Put her at once under chloroform. The next pain she did not lose consciousness, and it passed off in five minutes. The intermissions grew longer and pains shorter, till after eight hours ceased giving chloroform, having first given morphine and asafœtida.

She went full time, and was delivered, with no abnormal disturbance. Previous to giving chlorofoım no anodynes relieved her at all.

CASE XXIX. *Communicated by Prof. Austin Flint, Buffalo, N. Y.*

Mrs. I. B., aged 23 years. Prior to marriage had enjoyed good health. She became pregnant a month after marriage. In the early part of pregnancy she suffered much from nausea and vomiting. During the last two months she was greatly anasarcous. The urine was highly *albuminous.* The anasarca was some diminished after giving the bitart. potass.

She was confined on the 26th of Nov., 1856. The external parts of generation were much swelled from dropsical effusion, but the labor progressed rapidly and the delivery took place without trouble. The labor commenced at 6½, A. M., and was ended at 8, A. M. The patient took chloroform moderately during the labor.

Mrs. B. seemed quite comfortable until 12½, P. M., when she was seized with a convulsion while sleeping. It lasted two or three minutes. In an hour a second occurred. The paroxysms continued to occur at first pretty regularly after an hour's interval. They then became more frequent, recurring every 15 or 20 minutes, lasting from one to three minutes. They resembled in all points epileptic convulsions.

Prof. F. began the use of chloroform after the first convulsion, and kept her under its influence for several hours. This prevented him from knowing what would have been the mental condition in the interval. At length, the chloroform was omitted for some time. She was then restless and delirious between the paroxysms, attempting to get out of bed and requiring to be restrained. The use of the chloroform was resumed still more freely, and apparently postponed the paroxysm for an hour.

The embarrassment of breathing during the paroxysms was very great and stertor continued for some time after the convulsive movements ceased. Finally, the stertor continued constantly, and she expired directly after a severe paroxysm twelve and a-half hours after the first paroxysm. She had in all twenty-two paroxysms.

In addition to the chloroform, one drop of croton oil was administered. This was four hours before her death. No dejection followed. The loss of the power of deglutition prevented the repetition of this medicine. This constituted the treatment. The pulse had considerable volume and force.

Prof. James P. White saw the patient in consultation four hours before her death.

Case XXX. *Communicated to Committee, by Prof. James P. White, Buffalo, N. Y.*

1857, *June.* Mrs. W., the mother of several children, a large fleshy woman, but of an impaired state of health, so much so that she had been under medical treatment, more or less of the time for the previous year. Had been under Dr. White's care.

Being arrived at full period of gestation, on the evening of the 1st of June, she complained of headache, severe enough to lead to the apprehension of convulsions, although there was no increase of the pulse.

During the night labor came on, and was accomplished after a brief labor, without any unusual attendant circumstances, and Dr. W. left the patient at five o'clock, A. M., apparently doing well.

Within the next hour she was suddenly seized with convulsions. She was visited almost immediately, and found to be still in convulsions and *flowing* profusely. Some time elapsed before the uterine hæmorrhage was checked, and a large amount of blood was lost.

The administration of chloroform was immediately commenced and continued through the day, the inhalations being generally commenced at the approach of every convulsive seizure. Drop doses of croton oil were administered every one or two hours during the day.

Consciousness never returned; but towards nine o'clock, P. M., of the 2d, the convulsions ceased, the comatose condition remaining. During the day and night the bowels continued perfectly insensible to the impressions of croton oil, or of injections.

At 9, A. M., of the 3d, no very perceptible change, except that bowels were freely moving off. Unconscious, except that she would answer when loudly spoken to, but her replies furnished no connection with the questions put to her. At times restless. Chloroform not now given. No apparent change during the day.

On the marning of the 4th, no apparent change for the better, and no encouragement given to the husband to expect recovery; whereupon he proceeded to call a Botanic physician. The patient died in the course of the day, or day after.

The urine in this case was *albuminous*.

CASE XXXI. *Communicated to Committee, by Jas. S. Hawley, M. D., Buffalo, N. Y.*

*Dec.* 12, 1857. Mrs. Maynard, pregnant with her second child, had been suffering from slight labor pains for the last forty-eight hours. Had been visited by me on the evening of the 11th, previous to her confinement, but as her labor did not seem to have begun, I left her. She complained of constant thirst and frequent micturition. On the 12th reached the house about 8 o'clock, P. M., and found she had had two convulsions and was still unconscious from their effects. Her attendants informed me that she had complained of headache in the evening, and at the time of the convulsive attack was much congested about the head and face. Inferring from this description that the convulsions were apoplectic in their character, I determined to bleed her if my inference was sustained. On the occurrence of another convulsion, the appearances of congestion were not so well marked as I expected to see, still I proceeded to draw about ℥x of blood, when the state of the pulse admonished me to stop. Another convulsion followed in about fifteen minutes, when I administered half a grain of morphine, and sent to town, (about two miles,) for chloroform. The next convulsion was followed by another half grain of morphine. After this she remained fifty minutes without a convulsion, which was nearly three times the previous interval. Chloroform was now administered moderately, but the convulsions returned with increased evidence of cerebral congestion. Pulse 120, full and hard, and face flushed and hot. Another bleeding was resolved upon: sixteen ounces were taken. The convulsions not returning, the chloroform was not again used.

She had seven convulsions from 7½ to 11 o'clock. Up to the time the convulsions ceased, the labor had made no progress. The remainder of the labor was short and easy, and the patient was delivered of twins: the first at 4 o'clock, living, but very small; the second half an hour after, dead, but larger than the first.

Urine taken during the convulsions showed evident traces of *albumen;* that taken on the second day after exhibited no traces of it. She had taken cream of tartar for thirty-six hours previous to taking the second specimen. Recovery was favorable.

CASE XXXII. *Communicated by Prof. James P. White, of Buffalo, N. Y., to Committee.*

*Jan.* 10th, 1838. Mrs. H., aged 26 years, in her first pregnancy; seven months advanced. On the evening of the 9th was attacked with headache, and had some imperfection of vision, but not of so severe a character as to cause her to take to the bed.

Early on the morning of the 10th was attacked with convulsions, and Dr. Nott was called, and bled her freely. During the day she had eight convulsions; at [illegible] o'clock, P. M., when Dr. White visited her, he found her in complete coma; pulse 130; breathing stertorous; and bowels not been moved for three days; os uteri undilated; bladder distended, and urine, upon being drawn off by the catheter and tested, was found to be *albuminous.*

Before 8 o'clock, P. M., she had another convulsion, at which hour she was brought under the influence of the chloroform. Croton oil was administered every hour until copious dejections were procured. Messrs. Mason and Butler remained with her to administer the chloroform. She was kept moderately under its influence continuously, and when any manifestation of uneasiness, or indications towards a return of convulsions, the chloroform was increased. Until three o'clock, A. M., a period of seven hours, there had been no return of convulsions; during which time the bowels were freely moved. At this hour the condition of the patient being so promising chloroform was omitted, and the young gentlemen in attendance returned to their homes.

At 5 o'clock, A. M., labor was completed, Dr. Nott at the time being present, and a healthy male fœtus was born, accompanied by two convulsions rappidly succeeding each other. Now she was again put under the influence of chloroform, and its moderate impression maintained for several hours. There was no more return of the convulsions. Consciousness gradually returned during the day. A full opiate injection was administered towards the withdrawal of the chloroform.

Recovery from this time steadily progressed, and she was dismissed on the tenth day, at which time the albumen had disappeared from the urine.

Case XXXIII. *A primipara, aged fifteen years and nine months, delivered at full term, of twins, by the forceps, after having been about fifty hours in labor, Puerperal Convulsions having supervened; both infants living, and together with the mother, doing well.* In the practice of Dr. Gould. Operation by Prof. White. Case attended and reported by Benj. Heaton Lemon, M. D., Assistant Accoucheur to the "Buffalo Lying-in and Foundling Hospital of the Sisters of Charity."*

Maria Holland, first menstruated at the age of fourteen; the catamenia last appeared in the latter part of May, 1857. She has had good health during gestation, with the exception of swelling of the lower extremities for the last three weeks, which disappeared entirely just previous to labor.

She was taken in labor Thursday afternoon, Feb. 4th, and attended by Dr. Austin Flint, Jr., at the request of Dr. Gould, whose patient she was. No progress had been made, however, or was making.

Friday afternoon Dr. Gould saw her. Little progress; parts dry, and the os uteri rigid; exhibited nauseating doses of antimony.

At 7, P. M., I first saw her. Nausea was considerable, retching had occurred several times; the pains were frequent and apparently efficient; the mouth of the womb was dilated to about the size of half a dollar; the parts cool, moist, and soft.

At 12, P. M., she had frequent, strong bearing down pains; the "*os*" was dilated to about the size of half a dollar piece; the lips thin and membranous; the parts well lubricated and cool; considerable nausea.

Shortly after twelve the pains became less and less frequent and efficient; and at 1 o'clock, A. M., had ceased altogether; she slept till nearly 2 o'clock; on awaking she had a convulsion, followed in five minutes by another, more severe but shorter: scarcely ended, however, when another began. After an interval of three-quarters of an hour's rest, another convulsion; in five minutes more, another: these were of short duration. Chloroform was administered with the apparent effect of shortening and lessening the severity of the paroxysms. After an interval of fifteen minutes, a sixth convulsion, which was followed by three-quarters of an hour's calm and sleep, from which she was awakened by a seventh convulsion, followed in ten minutes by an eighth, when she rested half an hour. Each of the preceding parox-

* Buffalo Medical Journal, April, 1858, 679.

ysms lasted, about one and a-half minutes by the watch. A fit of three minutes' duration succeeded this rest, not as severe, apparently, though of longer duration than the preceding ones. Two more convulsions recurred at an hour's interval. From this time the patient was kept partially under the influence of chloroform.

She had twelve convulsions from 2 o'clock, A. M., up to 7, A. M. Between these hours she took two drachms of ergot infused in a pint of water. No perceptible effect was produced by it, however, being probably inert.

Under the partial influence of chloroform the patient remained passive, until the arrival of Prof. White and Dr. Newman, at half past 10, A. M.

Dr White drew off a small quantity of urine; ruptured the membranes; advised cold to the head, one drop of croton oil to be given immediately, and the continuance of the chloroform as before.

Dr. Newman, on testing the urine, *found no albumen.*

Shortly before twelve, slight pains were manifested at intervals. Dr. White pushed up the head in order that the waters might drain away with more facility. After this active labor set in, the pains being efficient and at regular intervals, so that by 2 o'clock, P. M., the vertex presented at the "os-externum," when the contractions again ceased, and the patient lay passive till half past two, when a convulsion succeeded.

Prof. White, at half past 3, P. M., delivered her under chloroform, with the forceps, of a live female child by the head; shortly after, with his hand of an asphyxiated female child, the feet presenting. Ergot was given, the secundines soon followed, and the womb contracted well.

The convulsions came on not so quickly but that the patient could give notice of it. They began first by severe pain in the head, then shaking of the extremities, and finally, the whole body becoming violently convulsed, the head thrown back, the jaws champing on a cork covered with a rag, to protect the tongue; foaming at the mouth; the jaws towards the close of the fit becoming *set* on the cork; the eyes wide, staring, and strabismic.

The paroxysm would terminate with sobbing and gasping; a deep stupor would follow, with stertorous respiration for some minutes, when the patient would recover sufficiently to speak. There average duration was about two minutes each, recurring at intervals of from five, ten, fifteen minutes, and an hour.

If chloroform was inhaled just previous to the access of the paroxysm, it would shorten and alleviate its severity if not prevent it entirely; little benefit seemed to be derived from it after the fit was on, respiration being partially suspended.

It may be worthy of remark, that in this case the convulsions twice supervened upon the cessation of labor pains; that the children were born alive, after the length of time in labor, with the addition of convulsions.

*No albumen* was found in the urine either before or after delivery.

The following case presents several points of interest; the uræmic symptoms being well marked, leading to apprehensions of convulsions; and as a preventive measure chloroform was employed. We have separated it from the other cases, and have not included it in our analysis, to avoid the objection which might be raised,—that the testimony which we have collected in the body of the report does not sustain the position of the *absolute* sequence and *invariable* development of convulsions upon the manifestation of albuminuric symptoms. This we shall not attempt to deny, or refute. At the same time, we would put the inquiry to any and every practitioner, whether he would not recognize in the symptoms developed in this case, every cause to anticipate an attack of convulsions during the course of labor; and if such an attack had been developed, whether he could have succeeded in framing an excuse which would have been received as sufficient by the profession, for neglecting to employ measures in anticipation of, or as preventive of an attack? And we would inquire farther, whether he would not under the circumstances, regard an escape from convulsions as a most happy deliverance?

CASE. *Communicated by P. H. Strong, M. D., Buffalo, N. Y.**

June 30th, 1857. I was called to attend Mrs. H., Niagara street, in her first confinement. She was of a frail, delicate constitution, and had marked œdema of the lower extremities and face; and which was also evident over the rest of her body, but less marked. She was pale and exsanguined; pulse about 100 and weak. She had for some time previously, been troubled with a good deal of *impairment of vision;* and also considerable *pain in region of kidneys.*

Her appearance at once suggested heart or kidney disease, one or both. An imperfect physical examination ef the heart, I thought revealed some degree of hypertrophy with slight effusion in pericardium.

As soon as practicable, and at an early stage of her labor, I subjected her urine to the heat test for *albumen*, which test revealed a deposit of a moderate amount of that product.

---

* See Buffalo Medical Journal, Aug., 1857, Transactions Buffalo Medical Association, p. 151.

With these indications, and superadded thereto the fair prospect from every source of a distressing and protracted labor, I became quite apprehensive of convulsions either during, or immediately succeeding labor.

The indications to my mind being imperative, to lighten the prospective heavy tax upon her very impressible nervous system, I resorted to *chloroform.*

Deeming it undesirable to produce complete anæsthesia, I strove for and obtained its partial, or benumbing effect with each pain; at no time producing complete unconsciousness. It produced in slight doses the happiest effects in allaying morbid sensibility, and in tranquilizing and sustaining her nervous system, and thus her muscular and vital forces.

*The result* was delivery after a labor of some ten hours, with no convulsions: though during the last hour, there was considerable involuntary nervous and muscular action of her system.

Her recovery was unusually protracted, but I believe is quite complete, though I have not examined her for kidney disease, or albuminuria, since the night of her labor.

An analytical examination of the foregoing cases of convulsions exhibits the following points of interest:

| | | | |
|---|---|---|---|
| The number of cases of convulsions collected, . . . | | | 33 |
| Of these there were: | Primiparæ, . . | 17 | |
| | Multiparæ, . . . | 9 | |
| | Not stated, . . | 7 | |
| Of the subjects of the convulsions there | | | |
| | Recovered, . . . | 24 | |
| | Died, . . . | 9 | |

The pathological symptoms and conditions are recorded as follows:

Described as being anasarcous, . . . . . 7 cases.

| | | | |
|---|---|---|---|
| The condition of the urine was noted in . . . | | | 19 cases. |
| Of these, it is described as being albuminous in . | | 12 | " |
| | Not albuminous in . | 2 | " |
| | *Dark colored* in . | 3 | " |
| | *No secretion* of urine, | 2 | " |

Attacks preceded by falls, or injuries, . . 2 cases.

*Points in reference to Treatment;* so far as noted in detail of the cases:

| | |
|---|---|
| *Bloodletting* was employed *before* the use of the anæsthetic agents, in . . . . . . . | 19 cases. |
| *Chloroform* was employed *previous* to bloodletting, or *without* venesection, in . . . . . . | 10 cases. |
| *Anæsthetics succeeded* by bleeding in . . . | 1 case. |
| *Marked effects* noted as *following* the use of *anæsthetics* in modifying and controlling convulsive movements in . . . . . . . | 23 cases. |
| The number in which chloroform did not diminish the convulsions, . . . . . . . . | 6 cases. |
| *Termination* of cases in which it is stated that *venesection* had been employed *previous* to administration of ether, or chloroform: | |
| No. of cases, . . . | 19 |
| Of these, there Recovered, . | 16 cases. |
| Died, . | 3 " |
| *Termination* of cases where *chloroform*, or *ether*, was administered *without* bloodletting being employed: | |
| No. of cases, . . . | 9 |
| Recovered, . . . | 6 |
| Died, . . . | 3 |
| Cases proving *fatal* where *chloroform* was administered, without previous treatment being indicated, . | 2 |
| No. of cases where chloroform, or ether having been administered at first with apparent benefit, subsequently appeared to lose their effects, other antispasmodics, (tinct. opii and asafœt.) were given: | |
| No. of cases, . . . . . | 3 |
| Recoveries, . . . | 2 |
| Died, . . . . . | 1 |

*Post-mortem* appearances in deaths following the administration of chloroform:

In two fatal cases following the use of chloroform, extravasation of blood within the brain or upon its surface, was found.

One case (xiii,) died a fortnight after the convulsions, and a *post-mortem* examination disclosed pneumonia; with healthy kidneys, brain and heart. I have called this case in this analysis a recovery from the convulsions.

*Prof. White* moved that the report be accepted and made the special order of business at the next regular meeting. This motion was seconded and carried

*Prof. Hamilton* presented a specimen of a tumor of the paroted gland, complete extirpation of she mass, including the entire gland; which is reported in full in another portion of the Journal.

The association then adjourned.

AUSTIN FLINT, Jr., M. D.,
Secretary *pro tem.*

